# SNRPA upregulation promotes mitochondrial function and drives CRPC aggressiveness

**DOI:** 10.1038/s41419-025-08302-8

**Published:** 2025-12-07

**Authors:** Xiao-long Liu, Lu Jin, Yong-qiang Yang, Mei-hua Lu, Bo-xin Xue

**Affiliations:** 1 Department of Urology, The Second Affiliated Hospital of Soochow University, Suzhou, China; 2 Department of Radiotherapy and Oncology, The Second Affiliated Hospital of Soochow University, Suzhou, China; 3 Department of Medical Records, The Second Affiliated Hospital of Soochow University, Suzhou, China

**Keywords:** Oncogenes, Prostate cancer

## Abstract

Identifying novel molecular targets for castration-resistant prostate cancer (CRPC) is crucial. This study examines the expression and functional role of small nuclear ribonucleoprotein polypeptide A (SNRPA), a core component of the U1 snRNP complex, in CRPC. Bioinformatics analyses indicate a positive correlation between SNRPA overexpression and the aggressiveness of prostate cancer, with high levels linked to poor outcomes. Single-cell RNA data further shows increased SNRPA expression in prostate cancer cells. Expression of SNRPA is also elevated in both locally-treated CRPC tissues and various CRPC cells. Knockdown via shRNA or knockout using CRISPR/Cas9 significantly reduced cellular proliferation, migration, and invasion in CRPC cells, while inducing apoptosis. SNRPA depletion decreased complex I activity, ATP production, and mitochondrial membrane potential, increased reactive oxygen species levels, and downregulated NDUFB8/NDUFS9 expression. In contrast, SNRPA overexpression enhanced the aggressive phenotype of CRPC cells, boosting mitochondrial complex I activity and ATP generation, while upregulating NDUFB8/NDUFS9. In vivo studies using xenograft models further validated the therapeutic potential of targeting SNRPA. SNRPA knockdown significantly inhibited CRPC xenograft growth, reduced ATP levels, and altered redox balance, as evidenced by decreased glutathione/glutathione disulfide ratio and increased lipid peroxidation. These effects were accompanied by decreased proliferation, increased apoptosis and downregulated NDUFB8/NDUFS9. Our findings collectively suggest that SNRPA plays a crucial role in driving CRPC progression and represents a promising therapeutic target.

## Introduction

Prostate cancer poses a major global health challenge, being the most frequently diagnosed cancer among men in developed countries [1, 2]. The emergence of castration-resistant prostate cancer (CRPC) presents a serious clinical issue due to its advanced progression and resistance to standard treatment options [3–5]. Although there have been notable advancements in the field, CRPC remains predominantly untreatable, highlighting the urgent need for innovative therapeutic approaches [3–5]. Current targeted therapies, including inhibitors of androgen receptor signaling and poly ADP-ribose polymerase, have demonstrated potential in extending survival [3–5]; however, they often face resistance. Identifying new molecular targets is essential for creating more effective and sustained treatments for patients with CRPC [3–5].

Small nuclear ribonucleoprotein polypeptide A (SNRPA) is an integral component of the U1 small nuclear ribonucleoprotein (snRNP) complex [6–8]. This complex plays a critical role in pre-messenger RNA (pre-mRNA) splicing, a fundamental step in mRNA maturation. SNRPA facilitates the precise removal of introns and subsequent exon ligation by binding to the 5’ splice site of pre-mRNA within the spliceosome machinery [6–8]. This accurate splicing process, guided by SNRPA, is essential for proper gene expression [6–8]. Dysregulation or mutations affecting SNRPA and other splicing factors have been linked to various human disease [6–8]. These findings underscore the crucial role of SNRPA in maintaining cellular function and integrity.

The precise role of SNRPA in tumorigenesis remains inadequately characterized. SNRPA has been shown to enhance tumor growth and progression possibly through its involvement in RNA splicing processes that affect gene expression [9–11]. For example, SNRPA promotes gastric cancer cell growth by modulating the expression of nerve growth factor (NGF) [11]. In clear cell renal cell carcinoma (ccRCC), high levels of SNRPA are associated with poor prognosis, as its overexpression leads to increased cell proliferation and tumor aggressiveness [9]. Jiang et al., reported that SNRPA acts as a key oncogenic gene and regulates key colorectal cancer-associated genes [10]. The present study investigated SNRPA expression and its potential functional significance in CRPC.

## Methods

### Reagents

Primary antibodies were sourced from Cell Signaling Technology (Danvers, MA). Viral constructs and validated mRNA primers were generously provided by Genechem (Shanghai, China). The CCK-8 cell viability assay kit, the antioxidant N-acetylcysteine (NAC), the synthetic androgen analog R1881 were obtained from Sigma Aldrich (St. Louis, MO). Promega (Madison, WI) supplied RNA-assay reagents, caspase inhibitors, the GSH/GSSG redox ratio kit, mitochondrial Complex I activity assay kit, and ATP assay kit. Fluorescent dyes were acquired from Thermo Fisher Scientific Invitrogen (Carlsbad, CA). The cell culture reagents and serum were provided by Corning Life Sciences (Corning, NY). The cell permeable ATP (ATP-polyamine-biotin) were purchased from MCE (Shanghai, China).

### Human cells

Primary human CRPC and epithelial cells were sourced from Dr. Mi [12]. Briefly, following surgical excision, tumor and adjacent epithelial tissues were mechanically dissociated and enzymatically digested using collagenase I and dispase II (Sigma). The resulting cell suspension was then cultured in complete medium, followed by a filtration step to enrich for adherent cell populations. Endothelial cells, fibroblasts, and immune cells and other non-adherent cells were discarded. Isolated primary cancer cells (designated “pPC-1”, “pPC-2”, “pPC-3” and “pPC-4”, derived from four patients) and epithelial cells (designated “pEpi1” and “pEpi2”, from two patients) were subsequently expanded using established culture protocols [12]. All procedures involving human cells were conducted with ethical approval from the Second Affiliated Hospital of Soochow University’s Ethics Committee, adhering to the principles outlined in the Declaration of Helsinki.

### Human tissues

This study included a cohort of fifteen (15) CRPC patients from the Affiliated Hospital of Jiangnan University, with ages ranging from 59 to 82 years, as reported previously [12]. Freshly procured CRPC tissues, along with paired normal prostate tissues adjacent to the tumor, were collected and cryopreserved in liquid nitrogen. An additional fifteen (15) castration-sensitive prostate cancer (CSPC) tissues from age-matched patients were also provided by Dr. Mi at the Affiliated Hospital of Jiangnan University [12]. Written-informed consent was obtained from all participants. These protocols were approved by the Ethics Committee of the Second Affiliated Hospital of Soochow University, ensuring adherence to the principles established in the Declaration of Helsinki.

### Western blotting

Briefly, protein lysates (20–35 µg) isolated from cells and tissues were subjected to sodium dodecyl sulfate-polyacrylamide gel electrophoresis using gels with a 10–12.5% polyacrylamide gradient. Following electrophoretic separation, proteins were transferred to polyvinylidene fluoride membranes. Membranes were subsequently blocked with a non-specific binding buffer and incubated overnight at 4 °C with primary antibodies targeting proteins of interest. After thorough washing steps, membranes were incubated with horseradish peroxidase-conjugated secondary antibodies for 45 min at room temperature. Protein bands were visualized using enhanced chemiluminescence (ECL) system, and band intensity was quantified using ImageJ software. The uncropped blotting images are listed in Fig. S1.

### Quantitative real-time PCR (qPCR)

Total RNA was extracted from cells and tissues using TRIzol reagents. Subsequently, the isolated RNA was reversely transcribed into complementary DNA using a commercially available Takara PCR amplification kit (Takara, Shiga, Japan). The resulting cDNA was then employed for qPCR analysis using SYBR Green PCR Master Mixes (Thermo-Fisher Invitrogen) on an ABI-7900 system. *Glyceraldehyde-3-phosphate dehydrogenase* (*GAPDH*) served as the reference gene for data normalization, and relative gene expression levels were calculated using the well-established 2^-ΔΔCt^ method.

### SNRPA knockdown

Two distinct short hairpin RNAs (shRNAs) targeting SNRPA were designed and cloned into the lentiviral vector GV369 (Genechem). The distinct shRNA sequences were: kdSNRPA-sh1and kdSNRPA-sh2. A scrambled control shRNA (“kdC”) was also included. Lentiviral production was achieved through co-transfection of these constructs with pMD2.G helper plasmids in HEK-293T cells. The resulting viral supernatants were employed to transduce primary cancer cells or epithelial cells. Following a 48-h incubation, selection for stable cells was performed using puromycin-containing medium. After five passages, stable cells with efficient SNRPA knockdown were established. Knockdown efficiency was subsequently validated using quantitative PCR (qPCR) and Western blotting analysis.

### SNRPA knockout (KO)

The primary CRPC cells or epithelial cells first achieved 50–60% confluence and were subsequently transduced with a lentiviral vector encoding Cas9 nuclease from Dr. Cao [13, 14] to establish stable expression. These cells were then transduced with a lentiviral construct designed for SNRPA knockout, obtained from Genechem. This construct expressed a single guide RNA (sgRNA) targeting a and verified specific sequence within SNRPA. Following puromycin selection to enrich for transduced cells, a single-cell cloning strategy was employed by plating cells at limiting dilution into 96-well plates. Successful SNRPA KO was verified through targeted deep sequencing of the sgRNA binding site and Western blotting analysis to confirm protein ablation. Two clones, designated “koSNRPA-SC1” and “koSNRPA-SC2”, exhibited complete depletion of SNRPA protein. Controls cells were transduced with an empty CRISPR/Cas9 lentiviral vector (“Cas9-C”) sourced also from Dr. Cao [13, 14].

### Gene overexpression

Overexpression of SNRPA, NDUFS8, NDUFS9 was induced in primary cancer cells using a lentiviral vector harboring the human *SNRPA*/*NDUFS8*/*NDUFS9* coding sequenceobtained from Genechem (Shanghai, China). A lentiviral vector lacking the coding sequence served as a control (“EV”). Lentiviral production was achieved by co-transfection of these constructs with helper plasmids into HEK-293T cells. The resulting viral supernatants were employed to transduce CRPC cells. Following a 36-h incubation, puromycin selection was employed to enrich for transduced cells. Stable cells exhibiting robust *SNRPA*/*NDUFS8*/*NDUFS9* overexpression were established after six passages. Overexpression efficiency was again validated using qPCR and Western blotting analyses.

### Cell viability and death assays

Briefly, following designated genetic treatments, 3000 cells per well were seeded in 96-well plates. After defined incubation periods, 20 µL/well of CCK-8 reagent was added and incubated for 90 min. Subsequent measurement of absorbance at 450 nm using a microplate reader provided a quantitative assessment of cell viability. Cell death was quantified using a Trypan blue staining kit (Biyuntian, Wuxi, China).

### Colony formation

Cells with the designated genetic modification were seeded at a density of 12,000 cells per well in 10-cm culture dishes containing complete medium supplemented with 10% FBS. To ensure optimal growth conditions, the medium was refreshed every 2 days. Following a 10-day incubation period, colonies were fixed, stained for visualization, and manually counted under a microscope.

### TUNEL/EdU nuclear staining

Cells subjected to designated treatments were seeded on coverslips placed within 24-well plates. Following defined incubation times, fixation was achieved with 4% formaldehyde for 8 min. Subsequently, cell membranes were permeabilized using 0.25% Triton X-100 in PBS for 4 minutes. Nuclei were then stained with either TUNEL for the specific detection of DNA fragmentation indicative of apoptosis, EdU to identify actively replicating cells, or DAPI for general nuclear visualization. Finally, fluorescent images were captured using a fluorescence microscope.

### Assessment of caspase activity

The enzymatic activities of caspase-3 and caspase-9 within the specified cell/tissue lysates were evaluated using a Caspase-3/-9 colorimetric assay kit procured from BioVision (Milpitas, CA). Active caspases in the lysate cleave the recognition sequences within these substrates, releasing chromophores detectable by absorbance measurement at 450 nm using a spectrophotometer (BioTek Instruments, Winooski, Vermont).

### Quantification of apoptosis by ELISA

Cells were seeded at a density of 3500 cells per well in 96-well plates and subjected to designated genetic modifications. Following incubation for specified durations, a quantitative ELISA assay (Millipore Sigma, Etobicoke, ON) was performed to measure histone-associated DNA fragments. The assay relied on a capture antibody specific to histone-DNA fragments within the samples. Subsequently, a colorimetric signal was generated at 450 nm upon enzymatic detection by a secondary antibody. The cytosol cytochrome C ELISA assays have been described in detail elsewhere [15, 16].

### Flow Cytometry for apoptosis detection

Cells with the applied genetic treatment were cultivated and were subjected to centrifugation to pellet the cells. The pelleted cells were then resuspended in the attached binding buffer (Sigma) and stained with Annexin V-APC (5 µL, Sigma) and/or propidium iodide (PI, 5 µL, Sigma). The stained cells were subsequently analyzed for apoptosis using a CytoFLEX flow cytometer (Beckman, Brea, CA). This involved the precise measurement of fluorescence signals, allowing for the differentiation between apoptotic (Annexin V positive) and non-apoptotic cells.

### Mitochondrial complex I activity and ATP quantification

Mitochondrial Complex I enzyme activity was determined in cellular and tissue lysates using a commercial colorimetric assay kit (Thermo Fisher Scientific Invitrogen, Carlsbad, CA). The assay is based on the spectrophotometric measurement of the conversion of NADH to NAD + . A decrease in absorbance at 360 nm was correlated to Complex I activity. Cell/tissue ATP levels were quantified using a Thermo Fisher Scientific Invitrogen colorimetric assay kit according to the manufacturer’s protocol. For each sample, a 30 μL aliquot containing 30 μg of total protein was utilized for analysis.

### Measuring the mitochondrial DNA contents

Mitochondrial DNA (mtDNA) contents were determined from total cellular lysates or fresh tissue samples. Total DNA was extracted using the phenol-chloroform method. Quantitative PCR (qPCR) was performed using primers targeting the mitochondrial gene *MT-ND1* (NADH dehydrogenase subunit 1) and the nuclear reference gene *GAPDH*. Relative mtDNA copy number was calculated using the ^ΔΔ^Ct method, normalizing *MT-ND1* Ct values to *GAPDH* Ct values. Standard cycling conditions were employed for all PCR reactions, and melt curve analysis confirmed primer specificity.

### Other fluorescence dye assays

CRPC cells were cultivated on coverslips and subjected to specific experimental conditions. Following the described incubation period, the cells were treated with designated fluorescent probes, including CellROX or JC-1. To eliminate unbound dye, cells were washed extensively with ice-cold phosphate-buffered saline (PBS). Fluorescence microscopy was executed using a Zeiss Axio Observer microscope, enabling the acquisition of fluorescence images. Subsequent to image capture, quantitative analysis of relative fluorescence intensity (RFI) was performed using ImageJ software.

### Lipid peroxidation assay

Lipid peroxidation was assessed using the thiobarbituric acid reactive substances (TBAR) assay. Tissue/cellular lysates, standardized to 30 μg of protein per sample, were analyzed using the TBAR assay kit (Cayman Chemical, Ann Arbor, MI). This kit quantifies malondialdehyde (MDA), a byproduct of lipid peroxidation. Lysates were incubated with thiobarbituric acid (TBA) at 95 °C for 60 min to form the MDA-TBA adduct, which was then measured spectrophotometrically at 550 nm. MDA levels were determined by comparison to a standard curve.

### GSH/GSSG detection

To quantify the ratio of reduced glutathione (GSH) to oxidized glutathione (GSSG), a commercially available GSH/GSSG ratio assay kit (Thermo-Fisher Scientific Invitrogen) was employed. Briefly, cellular or tissue lysates were incubated with 5,5’-dithiobis-(2-nitrobenzoic acid) (DTNB), glutathione reductase, and NADPH. Subsequently, the reaction mixture was added to the lysates, and absorbance at 425 nm was monitored spectrophotometrically over a five-minute interval. GSH and GSSG concentrations within the lysates were determined via interpolation from a standard curve. The calculated GSH/GSSG ratio was normalized to protein content for comparative analysis.

### In vivo xenograft studies

Five-week-old BALB/c nude mice (weight range: 17.8–18.3 g, all male) were obtained from SLAC (Shanghai Laboratory Animal Center, Shanghai, China) to establish an in vivo xenograft model. These mice received subcutaneous injections into their flanks with primary CRPC cells (six million cells per injection). Tumor development and subsequent growth were monitored starting at day 10 post-injection (“Day-10”). Tumor volumes, body weights, and daily tumor growth rate (mm³/day) were measured and calculated according to established protocols [12]. Xenograft tumors were excised, fixed in formalin, and embedded in paraffin. These sections underwent a standardized process encompassing deparaffinization, rehydration, and antigen retrieval. To minimize non-specific antibody binding, the sections were then blocked. Sections were then incubated with a primary antibody, followed by an HRP-conjugated secondary antibody. Visualization of the target protein was achieved through the enzymatic conversion of diaminobenzidine (DAB) by HRP, resulting in a brown precipitate. Additionally, sections were further stained using a commercially available TUNEL assay kit (Biyuntian, Wuxi, China) to evaluate apoptotic cell death or a Ki-67 fluorescence assay kit (Biyuntian, Wuxi, China) to evaluate proliferation within the tumors. Following thorough washing, these sections were counterstained with DAPI for nuclear identification. The sections were them mounted with an anti-fade mounting medium and examined under a fluorescence microscope for comprehensive analysis. The animal studies adhered to the guidelines set forth by the Institutional Animal Care and Use Committee (IACUC) and received ethical approval from the Ethics Review Board of the Second Affiliated Hospital of Soochow University.

### Statistics analyses

All in vitro experiments were performed in five replicates to ensure data robustness. Data normality was confirmed and results were presented as mean ± standard deviation (SD). Statistical analyses were conducted using SPSS software (SPSS Inc., Chicago, IL). For comparisons of two groups, an unpaired Student’s *t*-test was utilized. For comparisons involving three or more groups, a one-way analysis of variance (ANOVA) followed by post-hoc Scheffé's and Tukey’s tests were employed. A significance level of *P* < 0.05 was adopted throughout the study.

## Results

### Expression and prognostic value of *SNRPA* in prostate cancer

**Fig. 1:**
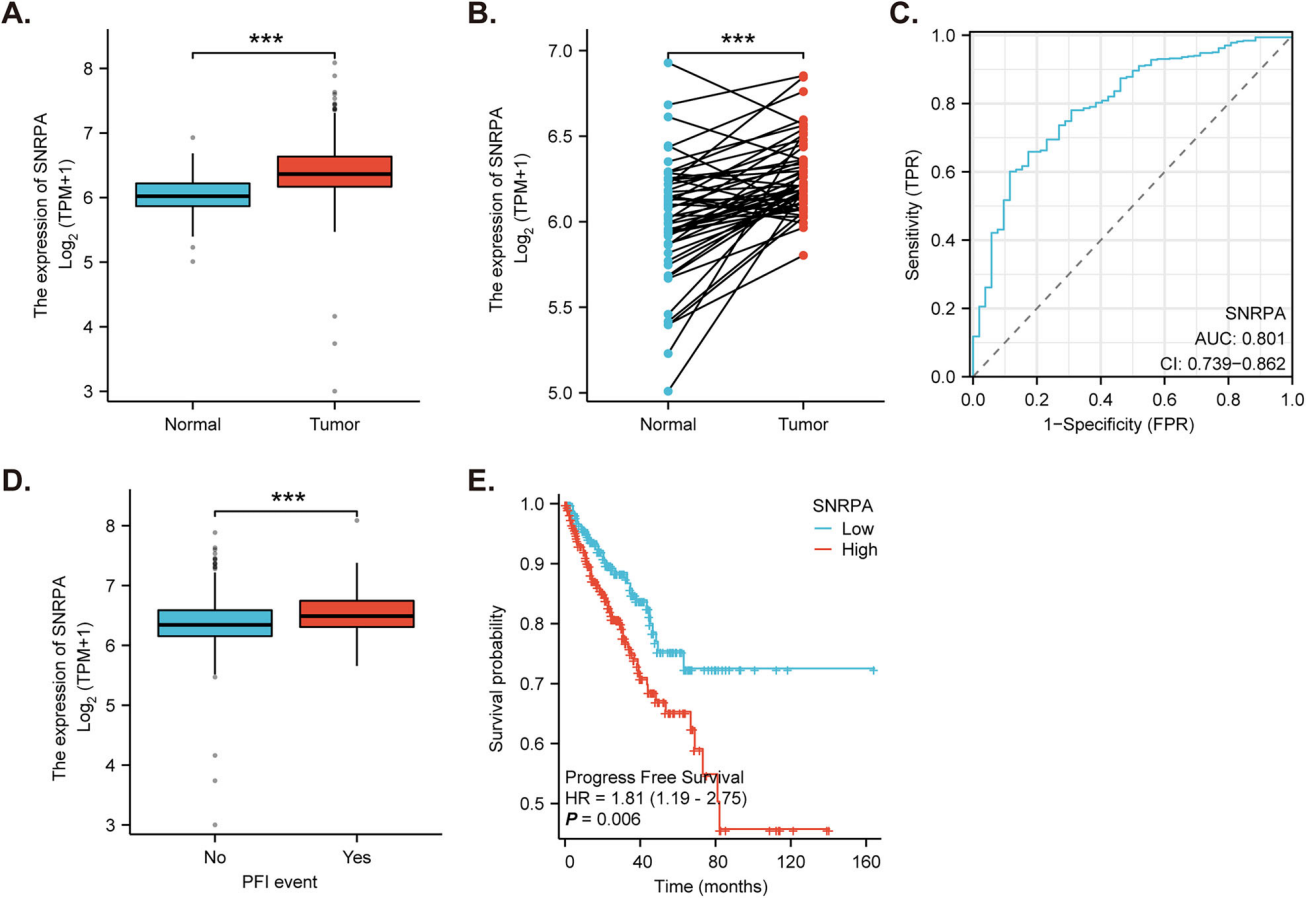
Expression and prognostic value of *SNRPA* in prostate cancer. Boxplot comparing *SNRPA* expression levels between normal and cancer prostate tissues from the Prostate Adenocarcinoma dataset within The Cancer Genome Atlas (TCGA-PRAD) (**A**). Scatter plot illustrating *SNRPA* expression in prostate tissues and paired surrounding normal prostate tissues (**B**). Receiver operating characteristic (ROC) curve analysis of *SNRPA* expression for differentiating between normal and cancer prostate tissues (**C**). Comparison of *SNRPA* expression between patients with and without a progression-free interval (PFI) event (**D**). Kaplan–Meier curves comparing progression-free survival (PFS) between patients with high and low *SNRPA* expression. Hazard ratio (HR) with 95% confidence interval (CI) is indicated (**E**). “TPM” stands for transcripts per million. “AUC” stands for area under the curve. “HR” stands for hazard ratio. “TPR” stands for true positive rate. “FPR” stands for false positive rate. ***P < 0.001.

The prostate adenocarcinoma dataset within The Cancer Genome Atlas (TCGA-PRAD) shows that *SNRPA* expression was significantly upregulated in prostate cancer tissues (“Tumor”) compared to normal prostate tissues (“Normal”) (Fig. 1A). In addition, paired tissue analysis showed that *SNRPA* expression was significantly increased in prostate cancer tissues (“Tumor”) compared to that in the paired normal prostate tissues (“Normal”) (Fig. 1B). Receiver operating characteristic (ROC) curve analysis revealed that *SNRPA* had excellent diagnostic efficiency in distinguishing between cancer and normal tissues, with an area under the curve (AUC) of 0.801 (95% CI: 0.739–0.862) (Fig. 1C). Additionally, *SNRPA* expression was significantly higher in patients who experienced a progression-free interval (PFI) event compared to those without (Fig. 1D). Furthermore, patients with high *SNRPA* expression exhibited significantly shorter progression-free survival (PFS) compared to those with low *SNRPA* expression (hazard ratio [HR] = 1.81, 95% CI: 1.19–2.75) (Fig. 1E). These findings collectively suggest that SNRPA overexpression might play a role in prostate cancer progression and may serve as a potential prognostic biomarker.

### Single cell RNA sequencing results shows upregulation of *SNRPA* in prostate cancer cells

**Fig. 2:**
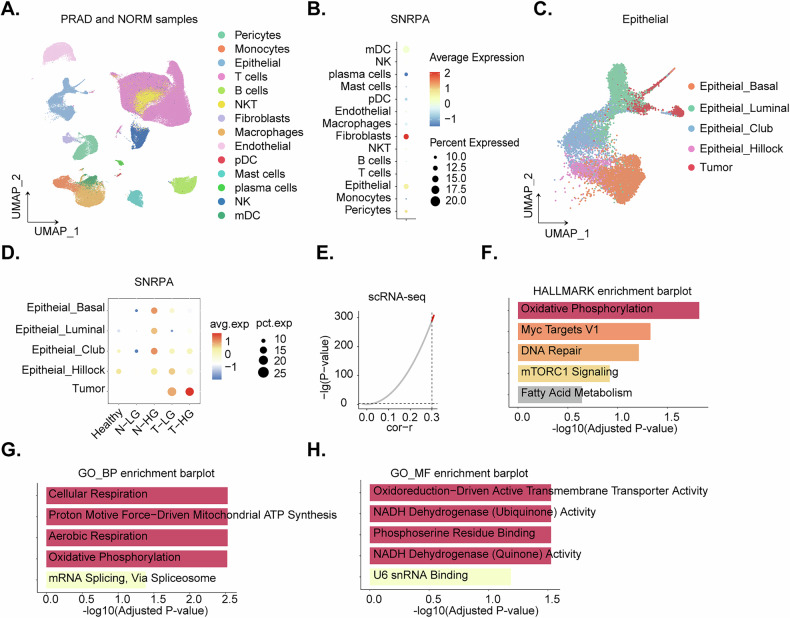
Single cell RNA sequencing shows upregulation of *SNRPA* in prostate cancer cells. UMAP dimensionality reduction of single cell RNA sequencing data from prostate tumor (“PRAD”) and paired cancer-adjacent normal prostate tissue (NORM) samples (**A**). Cell types are annotated by color (**A**). Dot plot showing *SNRPA* expression across different cell types (**B**). UMAP visualization of epithelial cell subclusters in PRAD (**C**). Dot plot showing *SNRPA* expression in epithelial cell subclusters in PRAD (**D**). Correlation analysis identified genes positively correlated with *SNRPA* expression (r > 0.3) in cancer epithelial cells of PRAD tissues (**E**). Gene set enrichment analysis (GSEA) enrichment plot for HALLMARK gene sets positively correlated with *SNRPA* expression in prostate cancer epithelial cells (**F**). Gene Ontology (GO) biological process (BP) and molecular function (MF) enrichment analyses for genes positively correlated with *SNRPA* expression in prostate cancer epithelial cells (**G**, **H**).

Single-cell RNA sequencing (scRNA-seq) data from the GEO dataset GSE181294 were analyzed to characterize cellular heterogeneity in prostate cancer. The dataset comprised tumor (“PRAD”), paired cancer-adjacent normal prostate tissue samples (“NORM”) (Fig. 2A). Cell type annotations were obtained from the original study [17]. Dimensionality reduction of the scRNA-seq data revealed distinct cellular clusters corresponding to annotated cell types (Fig. 2A). Dot plot analysis demonstrated that *SNRPA* expression was enriched in myeloid dendritic cells (mDCs), fibroblasts, and epithelial cells (Fig. 2B). To further investigate *SNRPA* expression in the epithelial compartment, we focused on this cell population and its subclusters (Fig. 2C). *SNRPA* expression was predominantly observed in tumor epithelial cells (“Tumor”) (Fig. 2C), with higher expression levels in high-grade tumors (“T-HG”) (Fig. 2D).

Correlation analysis identified genes positively correlated with *SNRPA* expression (r > 0.3) in epithelial cells (Fig. 2E). Gene set enrichment analysis (GSEA) using the HALLMARK gene sets revealed significant enrichment for oxidative phosphorylation (OXPHOS), MYC targets, DNA repair, and mTORC1 signaling pathways (Fig. 2F). Additionally, Gene Ontology (GO) biological process (BP) analysis indicated associations between *SNRPA* and cellular respiration, ATP synthesis, OXPHOS, and RNA splicing (Fig. 2G). GO molecular function (MF) analysis further suggested the possible involvement of *SNRPA* in oxidoreductase activity, transmembrane transporter activity, and NADH dehydrogenase activity (Fig. 2H. These scRNA-seq results again shows upregulation of *SNRPA* in prostate cancer cells.

### Elevated SNRPA expression in locally-treated CRPC tissues and primary human CRPC cells

**Fig. 3:**
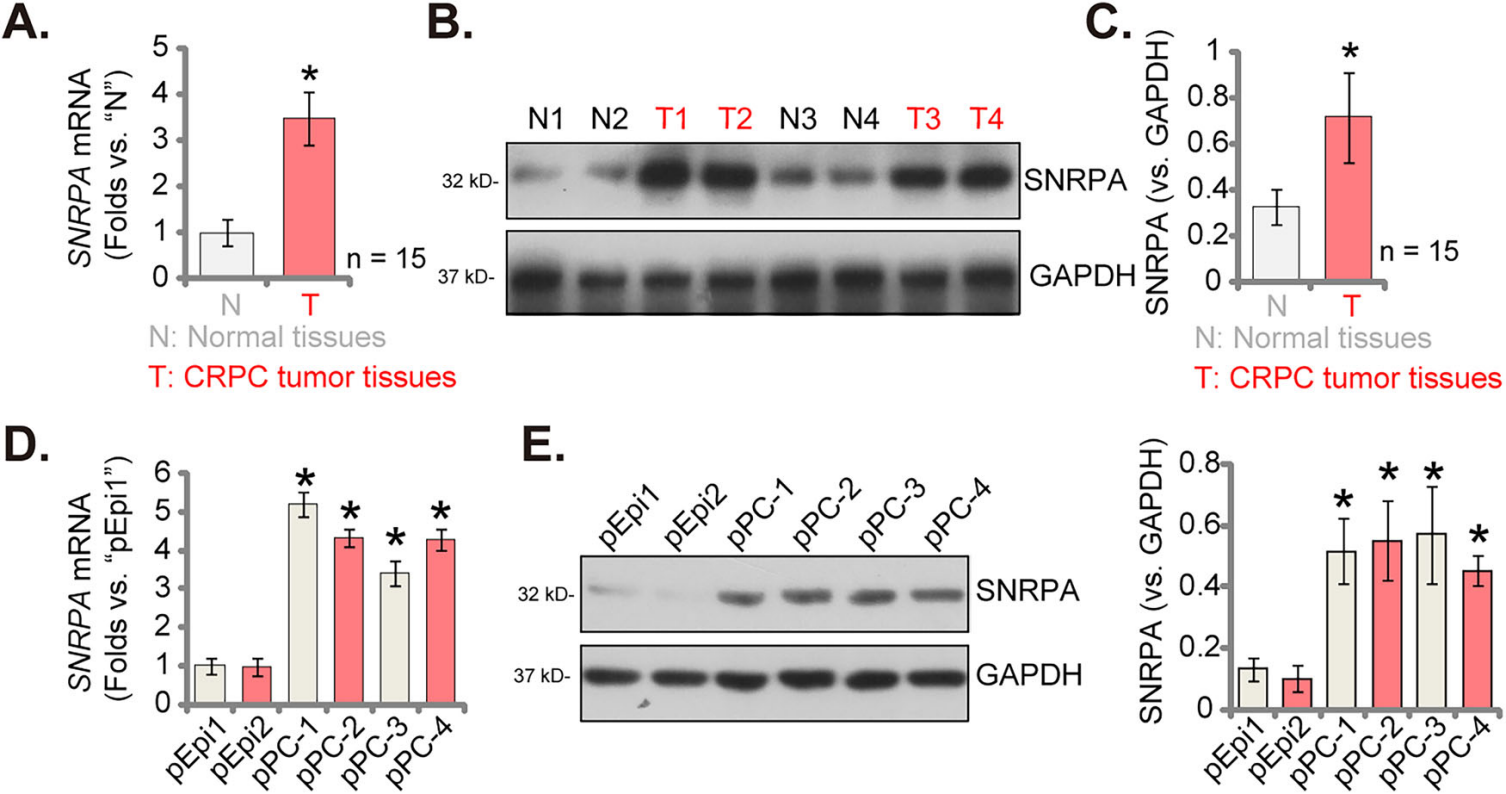
Elevated SNRPA expression in locally-treated CRPC tissues and primary human CRPC cells. *SNRPA* mRNA (**A**) and protein (**B** and **C**) levels in CRPC tissues (designated as “T”) obtained from 15 distinct patients were shown and these levels were compared to adjacent normal prostate tissues (designated as “N”). *SNRPA* mRNA (**D**) and protein (**E**) levels in primary CRPC cells (designated as “pPC-1” to “pPC-4”) derived from four patients, alongside primary human prostate epithelial cells (designated “pEpi-1” and “pEpi-2”) were shown. Data are presented as mean ± standard deviation (SD). Statistical significance is marked by * *P* < 0.05 when compared to “pEpi1” cells or “N” tissues.

We next investigated the potential role of SNRPA in CRPC by evaluating its mRNA and protein expression levels in tumor tissues. qPCR analysis revealed a significant upregulation of *SNRPA* mRNA in CRPC samples from 15 patients (as reported previously [12]) compared to adjacent normal prostate tissues (Fig. 3A). Western blotting analysis corroborated these findings, demonstrating elevated SNRPA protein levels in tumor samples from four representative patients (namely “T1”, “T2”, “T3”, and “T4”) (Fig. 3B). To evaluate the generalizability of the findings, Western blotting quantification was conducted on all 15 patient samples. This analysis revealed a statistically significant upregulation of SNRPA protein expression in CRPC tissues relative to paired normal samples. (*P* < 0.05, Fig. 3C). These findings suggest a potential role for SNRPA in CRPC development. We next analyzed its expression in primary CRPC cells derived from four patients (designated “pPC-1”, “pPC-2”, “pPC-3”, and “pPC-4”) [12]. qPCR revealed a statistically significant increase in *SNRPA* mRNA levels in all CRPC cells compared to primary human prostate epithelial cells (“pEpi-1” and “pEpi-2”) described previously [12] (Fig. 3D). This finding was corroborated by Western blotting analysis, demonstrating elevated SNRPA protein levels in all primary CRPC cells (Fig. 3E). Conversely, pEpi-1 cells and pEpi-2 cells exhibited low SNRPA protein expression (Fig. 3E).

### Silencing SNPRA impedes proliferation and migration in primary human CRPC cells

**Fig. 4:**
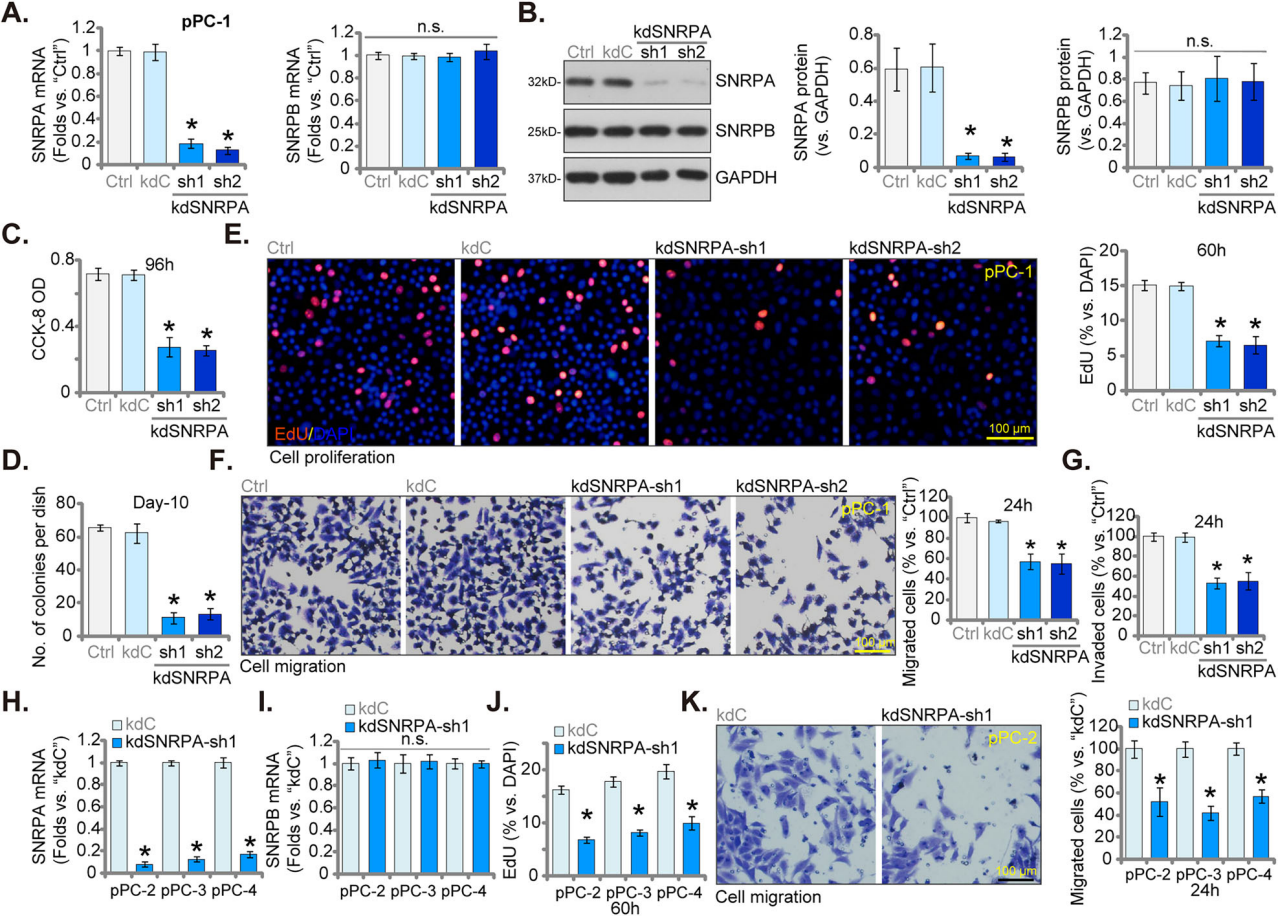
Silencing SNPRA impedes proliferation and migration in primary human CRPC cells. Primary human CRPC cells (pPC-1) cells were subjected to stable knockdown (kd) of SNRPA using lentiviral shRNAs, “kdSNRPA-sh1” and “kdSNRPA-sh2”, alongside a control non-targeting scrambled shRNA (“kdC”). Stable cell s were established following puromycin selection. Quantitative real-time PCR (qPCR) (**A**) and Western blotting (**B**) were performed to assess the efficiency of SNRPA knockdown. Equal numbers of pPC-1 cells expressing either kdC, kdSNRPA-sh1, or kdSNRPA-sh2 were cultured for defined time points. Subsequently, various cellular functions were evaluated, including cell viability (CCK-8 OD, **C**, colony formation (**D**), proliferation (tested by nuclear EdU incorporation, **E**, migration (tested by “Transwell” assay, **F**, and invasion (tested by “Matrigel Transwell” assay, **G**. Next, stable cells derived from independent primary CRPC cells (pPC-2, pPC-3, and pPC-4) were established, expressing either kdC or kdSNRPA-sh1. *SNRPA* and *SNRPB* mRNA expression levels were again evaluated using qPCR (**H**–**I**). These cells were cultured for defined time points, cell proliferation was measured using nuclear EdU incorporation assays (**J**) and migration was assessed via “Transwell” assays (**K**). Data are presented as mean ± standard deviation (SD) with n = 5 biological replicates. “Ctrl” denotes the parental control cells. Statistical significance is indicated by *P < 0.05 compared to “kdC” cells. “n.s.” denotes non-statistically significant differences (***P*** > 0.05). Consistent results were obtained across all five biological replicates. The scale bar in microscope images represents 100 μm.

To elucidate the functional significance of SNRPA in CRPC progression, we employed a lentiviral-mediated shRNA knockdown approach. Primary CRPC cells (“pPC-1”, established previously [12]) were transduced with lentiviral particles harboring SNRPA-specific shRNA sequences. Puromycin selection was employed to isolate stably transduced cells. Two independent shRNA constructs targeting SNRPA, designated “kdSNRPA-sh1” and “kdSNRPA-sh2,” were utilized. This strategy resulted in a significant downregulation of SNRPA at both the mRNA and protein levels, as demonstrated by qPCR and Western blotting assays (Fig. 4A–B). Notably, mRNA and protein expression of SNRPB, a related control gene, remained unaffected (Fig. 4A–B), indicating specific targeting of SNRPA by the applied shRNA constructs. To evaluate the impact of SNRPA silencing on CRPC cell viability, we employed the CCK-8 assay. Silencing SNRPA via the above shRNA constructs resulted in a significant decrease in pPC-1 cell viability (Fig. 4C). Furthermore, shRNA-mediated SNRPA knockdown significantly impeded pPC-1 cell proliferation, as demonstrated by decreased colony formation (Fig. 4D) and reduced EdU incorporation (Fig. 4E). Additionally, pPC-1 cell motility was demonstrably compromised by SNRPA depletion. “Transwell” and “Matrigel Transwell” assays revealed a substantial decrease in both in vitro migration and invasion capacities of pPC-1 cells following SNRPA knockdown (Fig. 4F–G). The control shRNA (kdC) did not exert any significant effects on SNRPA-SNRPB expression (Fig. 4A–B) or the functional properties (Fig. 4C–G) of pPC-1 cells, indicating the specificity of the shRNA approach.

To assess the generalizability of SNRPA knockdown effects, we transduced the lentiviral vector harboring kdSNRPA-sh1 into additional primary human CRPC cells (“pPC-2”, “pPC-3”, and “pPC-4” [12]). Stable selection was achieved using puromycin. Consistent with observations in pPC-1 cells, kdSNRPA-sh1 treatment resulted in a significant downregulation of *SNRPA* mRNA in all three additional CRPC cells (Fig. 4H), with no alterations detected in *SNRPB* mRNA levels (Fig. 4I). Furthermore, mirroring the effects in pPC-1 cells, kdSNRPA-sh1 significantly impaired cell proliferation (as measured by EdU incorporation, Fig. 4J) in all the additional primary CRPC cell lines. kdSNRPA-sh1 also significantly diminished cell migration in these diverse CRPC cells (Fig. 4K). Together, these results showed that SNPRA shRNA impeded proliferation and migration in primary human CRPC cells.

### Silencing SNPRA induces apoptosis in primary human CRPC cells

**Fig. 5:**
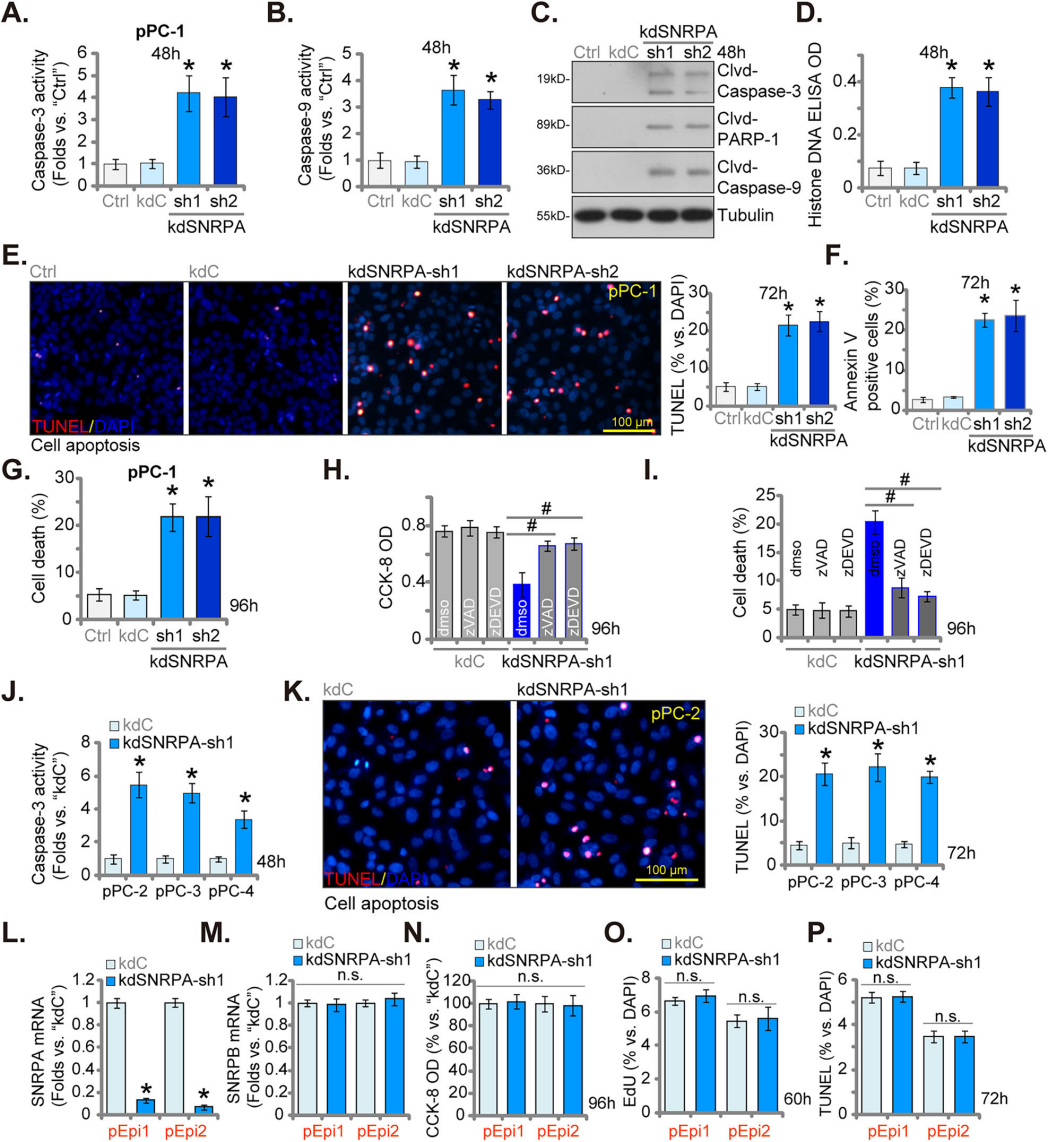
Silencing SNPRA induces apoptosis in primary human CRPC cells. Primary human CRPC cells (pPC-1) cells were subjected to stable knockdown (kd) of SNRPA using lentiviral shRNAs, “kdSNRPA-sh1” and “kdSNRPA-sh2”, alongside a control non-targeting scrambled shRNA (“kdC”). Stable cells were established following puromycin selection, cells were cultured for defined time points, the Caspase-3/Caspase-9 activities (**A**–**B**), expression of apoptosis-associated proteins (**C**), and the Histone-bound DNA contents (**D**) were tested, and cell apoptosis measured via nuclear TUNEL staining (**E**) and Annexin V-PI FACS (**F**) assays. Cell death was quantified via measuring Trypan blue-positive cells (**G**). pPC-1 cells expressing either kdC or kdSNRPA-sh1 were treated with zVAD-fmk (zVAD, 50 μM), zDEVDfmk (zDEVD, 50 μM) or vehicle control (0.1% DMSO, “dmso”) for designated hours, cell viability (CCK-8 OD, **H**) and death (Trypan blue uptake, **I**) were measured. Additionally, stable cells derived from other primary CRPC cells (pPC-2, pPC-3, and pPC-4) (**J** and **K**) as well as the primary human prostate epithelial cells (pEpi1 and pEpi2) (**L**–**P**) expressing either “kdC” or “kdSNRPA-sh1” were established and *SNRPA*/*SNRPB* mRNA expression was examined (**L** and **M**); Equal cell numbers were cultured for designated time periods, the Caspase-3 activity (**J**), cell apoptosis (by measuring TUNEL-nuclei ratio, **K** and **P**), viability (CCK-8 OD, **N**) and proliferation (by measuring EdU-nuclei ratio, **O**) were measured similarly. Data are presented as mean ± standard deviation (SD) with n = 5 biological replicates. “Ctrl” denotes the parental control cells. Statistical significance is indicated by *P < 0.05 compared to “kdC” cells. ^#^***P*** < 0.05 (**H** and **I**). “n.s.” denotes non-statistically significant differences (*P* > 0.05). Consistent results were obtained across all five biological replicates. The scale bar in microscope images represents 100 μm.

To gain deeper insights into the functional consequences of SNRPA silencing, we explored its influence on cell death mechanisms. Our findings revealed an increase in Caspase-3 and Caspase-9 activities in pPC-1 cells transfected with kdSNRPA-sh1 or kdSNRPA-sh2 shRNAs (Fig. 5A–B). Consistent with the observed caspase activity increase, shRNA-mediated SNRPA knockdown induced the cleavage of Caspase-3, Caspase-9, and Poly(ADP-ribose) polymerase 1 (PARP-1), hallmarks of the apoptotic execution pathway (Fig. 5C). Additionally, we observed elevated levels of histone-bound DNA, a well-established marker of apoptosis initiation, in pPC-1 cells expressing SNRPA-targeting shRNA (Fig. 5D). These data suggest that SNRPA knockdown triggers a cascade of apoptotic events, culminating in heightened apoptosis in pPC-1 cells. This is further corroborated by the substantial increase in TUNEL-positive nuclei (Fig. 5E) and a heightened percentage of Annexin V-positive cells (Fig. 5F) following SNRPA knockdown. Control shRNA (kdC) treatment did not induce apoptosis in pPC-1 cells (Fig. 5A–F), highlighting the specificity of SNRPA silencing in promoting CRPC cell apoptosis. Concordant with the induction of apoptosis markers, SNRPA knockdown using targeted shRNA resulted in a significant increase in pPC-1 cell death, as demonstrated by enhanced Trypan blue uptake (Fig. 5G). Notably, both the Caspase-3 specific inhibitor zDEVD-fmk and the pan-caspase inhibitor zVAD-fmk significantly abrogated the reduction in cell viability (Fig. 5H) and cell death (Fig. 5I) observed in pPC-1 cells transfected with kdSNRPA-sh1. These findings strongly suggest that caspase-dependent apoptosis is the principal mechanism underlying SNRPA silencing-mediated cytotoxicity in primary CRPC cells.

We also investigated the effect of SNRPA knockdown in additional primary human CRPC cells (“pPC-2”, “pPC-3”, “pPC-4”). Stable SNRPA silencing was established using the previously described kdSNRPA-sh1 lentiviral vector (see Fig. 4). Consistent with our observations in pPC-1 cells, this approach resulted in a significant increase in Caspase-3 activity (Fig. 5J) across all three additional CRPC cells. Furthermore, a marked elevation in the number of TUNEL-positive nuclei was observed in these cells (Fig. 5K), signifying widespread induction of apoptosis following SNRPA depletion. To evaluate the specificity of the observed effects, we investigated the impact of SNRPA knockdown on primary human prostate epithelial cells (“pEpi1” and “pEpi2”) [12]. Treatment with the kdSNRPA-sh1 lentiviral vector resulted in a significant reduction in *SNRPA* mRNA levels within these cells (Fig. 5L), with no discernible changes observed in *SNRPB* mRNA expression (Fig. 5M). Importantly, unlike the observed cytotoxicity in CRPC cells, SNRPA silencing did not alter cell viability as measured by CCK-8 assay (Fig. 5N) or cell proliferation as assessed by nuclear EdU incorporation (Fig. 5O) in these non-malignant epithelial cells. Furthermore, no evidence of apoptosis induction was observed through TUNEL staining assays in pEpi1 and pEpi2 cells following SNRPA silencing (Fig. 5P). These findings suggest a selective cytotoxic effect of SNRPA knockdown in CRPC cells, highlighting its potential as a targeted therapeutic strategy.

### SNRPA knockout suppresses proliferation, migration, and induces apoptosis in primary human CRPC cells

**Fig. 6:**
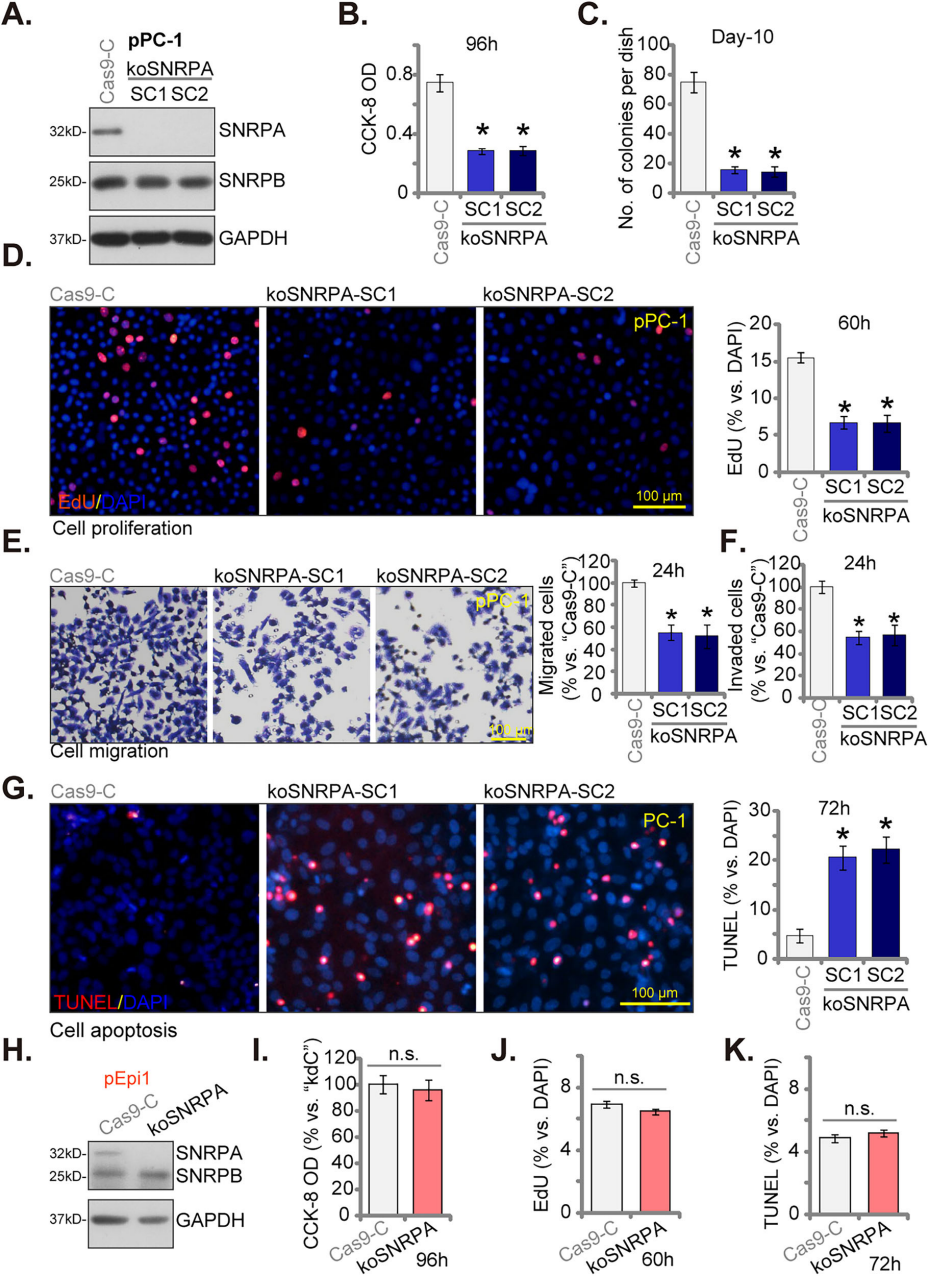
SNRPA knockout suppresses proliferation, migration, and induces apoptosis in primary human CRPC cells. Cas9-expressing pPC-1 cells were transduced with either a CRISPR/Cas9-SNRPA-KO construct targeting small guide RNA of SNRPA or a control vector (“Cas9-C”). Subsequently, single-cell derived colonies were isolated from the KO groups, designated koSNRPA-SC1 and koSNRPA-SC2 (two stable colonies, “SC”). Western blotting confirmed specific depletion of SNRPA protein in koSNRPA cells (**A**). The above cells were cultured for defined time points and various cellular functions were evaluated, including cell viability (CCK-8 OD, **B**), colony formation (**C**), proliferation (tested by nuclear EdU incorporation, **D**), migration (tested by “Transwell” assay, **E**), and invasion (tested by “Matrigel Transwell” assay, **F**) were measured. Cell apoptosis was tested via TUNEL staining assays (**G**). Cas9-expressing pEpi-1 prostate epithelial cells were transduced with either the CRISPR/Cas9-SNRPA-KO construct (“koSNRPA”) or “Cas9-C”, and single stable cells obtained, expression of SNRPA and SNRPB proteins was shown (**H**); The above cells were cultured for defined time points, cell viability (CCK-8 OD, **I**), proliferation (tested by nuclear EdU incorporation, **J**) were measured, with cell apoptosis tested via quantifying TUNEL-positive nuclei ratio (**K**). Data are presented as mean ± standard deviation (SD) with n = 5 biological replicates. Statistical significance is indicated by *P < 0.05 compared to “Cas9-C” cells. “n.s.” denotes non-statistically significant differences (***P*** > 0.05). Consistent results were obtained across all five biological replicates. The scale bar in microscope images represents 100 μm.

To circumvent potential off-target effects inherent to shRNA approaches and achieve definitive SNRPA ablation, we employed CRISPR/Cas9 technology. A lentiviral vector expressing a small guide RNA targeting *SNRPA* (CRISPR/Cas9-SNRPA-KO) were transduced into Cas9-pre-expressing pPC-1 cells, along with a puromycin resistance gene for selection. Following puromycin treatment, isolated clones underwent rigorous screening for complete *SNRPA* knockout. This process yielded single-cell derived pPC-1 colonies exhibiting SNRPA deficiency, designated as “koSNRPA-SC1” and “koSNRPA-SC2”. Western blotting analysis confirmed complete loss of SNRPA protein expression in these KO cells (Fig. 6A), whereas SNRPB protein levels remained unchanged (Fig. 6A), signifying the specificity of the CRISPR/Cas9-mediated KO strategy. Mirroring observations in SNRPA-silenced cells, CRISPR/Cas9-induced SNRPA KO resulted in diminished cell viability (measured by CCK-8 OD, Fig. 6B) and inhibited colony formation (Fig. 6C). pPC-1 cell proliferation was robustly suppressed by SNRPA KO, evidenced by a significant decrease in EdU-positive nuclei (Fig. 6D). Furthermore, “Transwell” assays revealed that koSNRPA-SC1 and koSNRPA-SC2 pPC-1 cells displayed a markedly reduced capacity for both in vitro migration (Fig. 6E) and invasion (Fig. 6F). Additionally, SNRPA KO triggered an apoptotic response in pPC-1 cells, as demonstrated by an elevated nuclear TUNEL ratio (Fig. 6G). Taken together, these findings reiterate the critical role of SNRPA in regulating essential cellular processes in CRPC cells.

To investigate the functional significant of SNRPA in non-cancerous prostate epithelial cells, CRISPR-Cas9-mediated KO was employed in the primary human prostate epithelial cells “pEpi1”. Western blotting analysis confirmed successful depletion of SNRPA protein expression in the resulting koSNRPA pEpi1 cells (Fig. 6H). Unlike the observed effects in CRPC cells, SNRPA KO did not significantly impact cell viability (measured by CCK-8 assay, Fig. 6I) or proliferation (assessed by EdU incorporation, Fig. 6J). Additionally, SNRPA KO did not induce apoptosis activation in pEpi1 cells (Fig. 6K). These findings suggest a specific role for SNRPA in CRPC cell progression.

### SNRPA overexpression enhances proliferation and migratory capacity in primary human CRPC cells

**Fig. 7:**
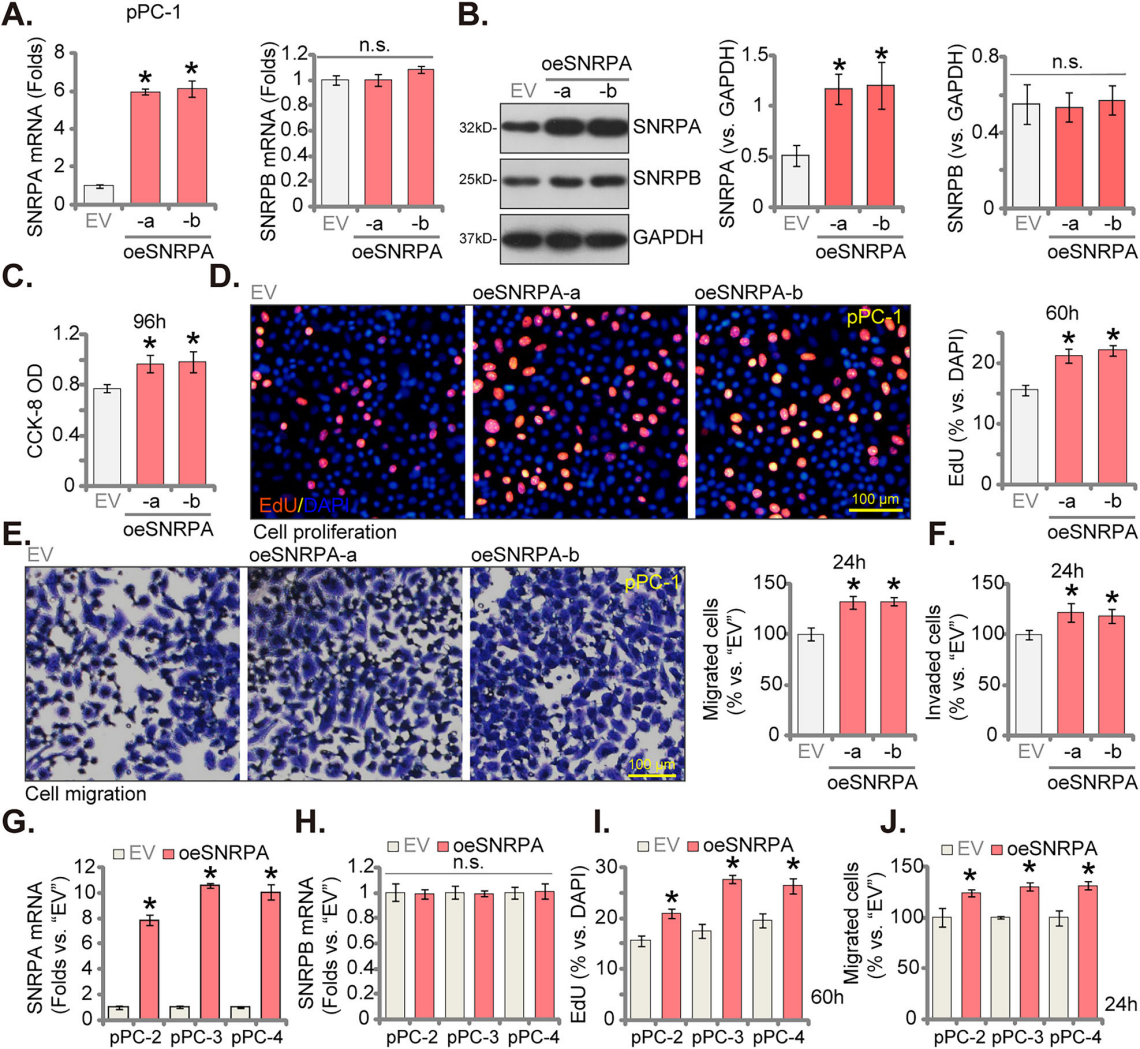
SNRPA overexpression enhances proliferation and migratory capacity in primary human CRPC cells. pPC-1 cells were infected with a lentiviral vector carrying the SNRPA-expressing sequence, creating two stable cell selection (“oeSNRPA-a” and “oeSNRPA-b”), an empty vector transduced the control cells (designated “EV”); Expression of SNRPA and SNRPB was tested (**A**–**B**). Cells were cultured for defined time points, and various cellular functions were evaluated, including cell viability (CCK-8 OD, **C**), proliferation (tested by nuclear EdU incorporation, **D**), migration (tested by “Transwell” assay, **E**), and invasion (tested by “Matrigel Transwell” assay, **F**). Primary human CRPC cells from three patients (pPC-2, pPC-3, and pPC-4) were similarly engineered with the SNRPA-expressing lentivirus (“oeSNRPA”) or the empty vector (“EV”). *SNRPA* and *SNRPB* mRNA levels were measured (**G**–**H**). Cells were cultured for defined time points, cell proliferation (**I**) and migration (**J**) were evaluated using the same methods as for pPC-1 cells. Data are presented as mean ± standard deviation (SD) with *n* = 5 biological replicates. Statistical significance is indicated by *P < 0.05 compared to “EV” cells. “n.s.” denotes non-statistically significant differences (*P* > 0.05). Consistent results were obtained across all five biological replicates. The scale bar in microscope images represents 100 μm.

Inhibition of SNRPA expression, by shRNA or CRISPR/Cas9 method, has been shown to exert significant anti-CRPC cell effects. To elucidate the opposing effects of enhanced SNRPA expression, we employed a lentiviral vector to introduce a construct encoding SNRPA into primary pPC-1 cells. Following transduction, puromycin selection was employed to isolate stably transduced cells. The resultant cell populations, designated “oeSNRPA-a” and “oeSNRPA-b”, were subsequently evaluated for SNRPA expression. Quantitative analysis revealed a significant elevation in both *SNRPA* mRNA (Fig. 7A) and protein (Fig. 7B) levels in oeSNRPA-a and oeSNRPA-b pPC-1 cells compared to empty vector (“EV”) control pPC-1 cells. *SNRPB* mRNA and protein expression remained unaffected (Fig. 7A–B). Functional assays revealed a significant enhancement in cell viability of SNRPA-overexpressing pPC-1 cells, as measured by the CCK-8 assay (Fig. 7C). This overexpression further promoted cell proliferation, supported by an elevated ratio of EdU-positive nuclei (Fig. 7D). Additionally, SNRPA overexpression significantly stimulated in vitro cell motility (Fig. 7E) and invasive potential (Fig. 7F) of pPC-1 cells. In other primary cells pPC-2, pPC-3, and pPC-4, stable transduction with the lentiviral SNRPA construct resulted in a marked upregulation of *SNRPA* mRNA levels (denoted as “oeSNRPA”, Fig. 7G), with no observed changes in *SNRPB* mRNA expression (Fig. 7H). Consistent with the findings in pPC-1 cells, SNRPA overexpression enhanced cellular proliferation, as indicated by increased EdU incorporation (Fig. 7I), and promoted in vitro cell motility (Fig. 7J) in these additional primary CRPC cells. Collectively, these observations again highlight the critical role of SNRPA in driving aggressive cellular phenotypes in CRPC.

### SNRPA is important for OXPHOS and ATP synthesis in primary human CRPC cells

**Fig. 8:**
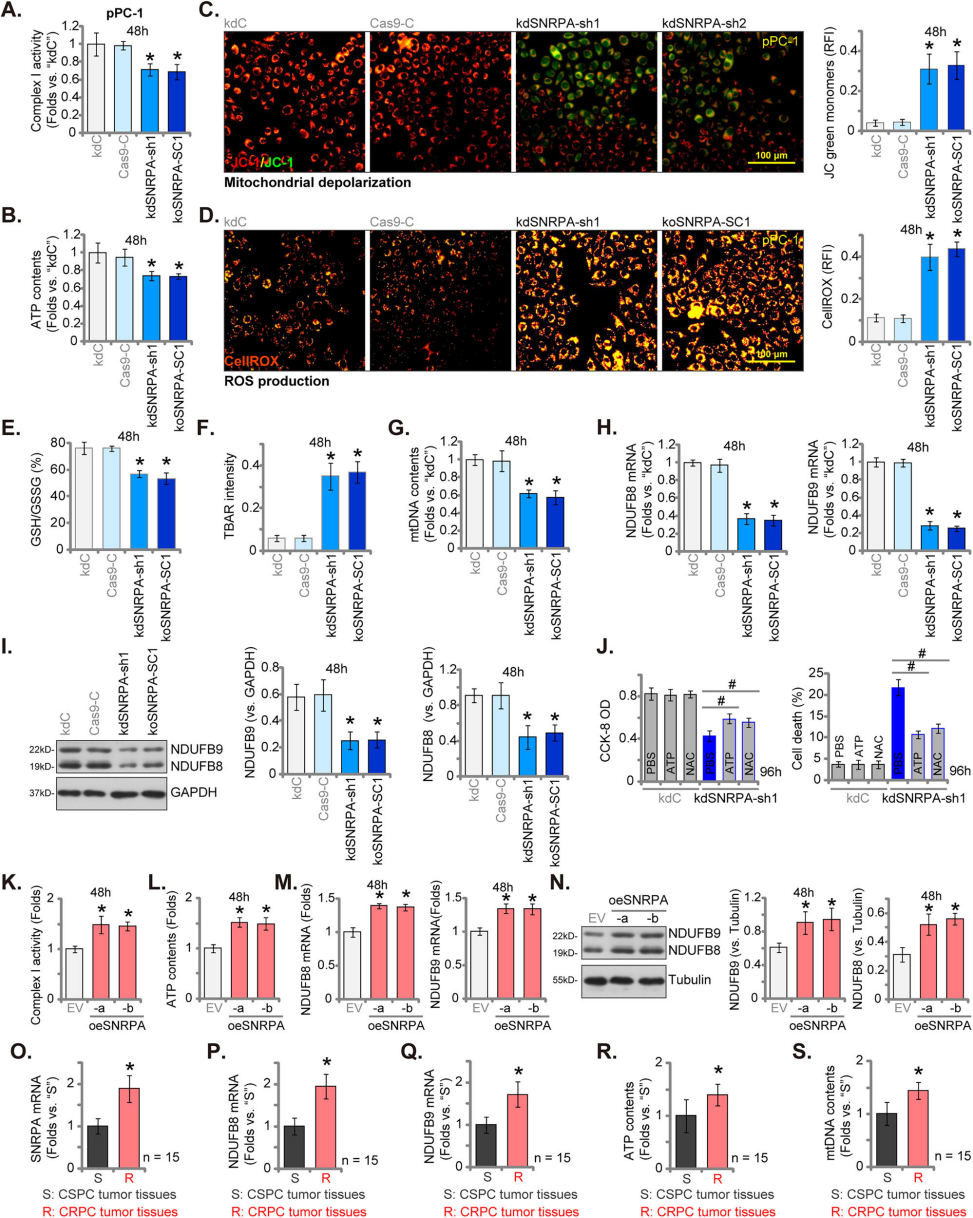
SNRPA is important for OXPHOS and ATP synthesis in CRPC cells. Primary human CRPC cells (pPC-1) with SNRPA shRNA (“kdSNRPA-sh1”), a control non-targeting scrambled shRNA (kdC), a CRISPR/Cas9-SNRPA-KO construct targeting small guide RNA of SNRPA (“koSNRPA-SC1”) or a control vector (“Cas9-C”) were cultured for defined time points, the mitochondrial complex I activity (**A**) and ATP contents (**B**) were measured; Mitochondrial depolarization (JC-1 monomers intensity, **C**), ROS production (CellROX intensity, **D**), GSH/GSSG ratio (**E**), lipid peroxidation (TBAR intensity, **F**) and mtDNA contents (**G**) were tested as well. Expression of NDUFB8/NDUFB9 was also shown (**H**–**I**); pPC-1 cells expressing either kdC or kdSNRPA-sh1 were treated with ATP (10 mM), NAC (500 μM) or vehicle control (PBS) for 96 h, cell viability (CCK-8 OD) and death (Trypan blue uptake) were measured (**J**). pPC-1 cells with overexpressed SNRPA (“oeSNRPA-a” and “oeSNRPA-b”) or an empty vector (designated “EV”) were cultured for defined time points, the mitochondrial complex I activity (**K**), ATP contents (**L**) and NDUFB8/NDUFB9 expression (**M**–**N**) were measured. CRPC tissues obtained from 15 human patients (*n* = 15) and the another castration-sensitive prostate cancer (CSPC) tissues from 15 age-matched patients (*n* = 15) were obtained, expression of listed mRNAs were tested (**O**–**Q**); ATP contents (**R**) and mtDNA contents (**S**) were also measured. Data are presented as mean ± standard deviation (SD) with *n* = 5 biological replicates (**A**–**N**). Statistical significance is indicated by *P < 0.05 compared to “kdC” cells or “EV” cells (**A**–**N**). *P < 0.05 compared to CSPC tissues (**O**–**S**). ^#^***P*** < 0.05 (**J**). Consistent results were obtained across all five biological replicates for in vitro studies. The scale bar in microscope images represents 100 μm.

The bioinformatics analyses (Fig. 2) suggested a positive correlation between *SNRPA* expression and genes involved in oxidative phosphorylation (OXPHOS) and ATP synthesis in prostate cancer cells. We next assessed OXPHOS and ATP levels in SNRPA-depleted pPC-1 cells. As shown, both SNRPA knockdown (kdSNRPA-sh1) and knockout (koSNRPA-SC1) resulted in a significant decrease in mitochondrial complex I activity (Fig. 8A) and ATP contents (Fig. 8B) in pPC-1 cells. Moreover, SNRPA depletion induced mitochondrial depolarization, as evidenced by increased JC-1 green monomers’ accumulation (Fig. 8C). Consistent with mitochondrial dysfunction, SNRPA-deficient pPC-1 cells exhibited elevated ROS levels, as indicated by enhanced CellROX intensity (Fig. 8D) and decreased glutathione (GSH)/oxidized glutathione (GSSG) ratio (Fig. 8E). Further supporting oxidative stress, lipid peroxidation was increased in SNRPA-depleted pPC-1 cells, as determined by elevated TABR intensity (Fig. 8F).

Importantly, the mtDNA contents (Fig. 8G) as well as mRNA and protein expression of two critical subunits of mitochondrial complex I, NDUFB8 and NDUFB9, were downregulated in SNRPA-deficient pPC-1 cells (Fig. 8H–I). To further elucidate the underlying mechanisms of SNRPA depletion-induced anti-CRPC cell activity, we investigated the effects of ATP supplementation and antioxidant treatment. Supplementation with ATP or the antioxidant N-acetylcysteine (NAC) significantly attenuated the decrease in cell viability and cell death (Fig. 8J) observed in kdSNRPA-sh1 pPC-1 cells. In contrast, the Caspase-3 specific inhibitor zDEVD-fmk and the pan-caspase inhibitor zVAD-fmk failed to restore the mitochondrial Complex I activity, ATP contents and mtDNA contents in pPC-1 cells with kdSNRPA-sh1 (Fig. S2A–C). These findings strongly suggest that impaired OXPHOS/ATP synthesis and subsequent oxidative stress are primary contributors to SNRPA silencing-induced apoptosis in primary human CRPC cells.

To rescue the phenotype, kdSNRPA-sh1-expressing pPC-1 cells were treated with co-added NDUFS8-expressing and NDUFS9-expressing lentiviral constructs (“oeNDUFS8/9”). This treatment successfully restored *NDUFS8* and *NDUFS9* mRNA expression (Fig. S2D–E) while leaving *SNRPA* mRNA levels unaffected (Fig. S2F) in pPC-1 cells. Importantly, the oeNDUFS8/9 treatment largely reversed the functional consequences of kdSNRPA-sh1, including reduced ATP (Fig. S2G), inhibited proliferation (Fig. S2H), and increased apoptosis (Fig. S2I).

Overexpression of SNRPA in pPC-1 cells (oeSNRPA-a and oeSNRPA-b, see Fig. 7) resulted in increased mitochondrial complex I activity (Fig. 8K) and ATP contents (Fig. 8L). Moreover, the mRNA (Fig. 8M) and protein (Fig. 8N) expression levels of NDUFB8 and NDUFB9 were upregulated. These results supported that SNRPA is important for NDUFB8/NDUFB9 expression, OXPHOS process and ATP synthesis in primary human CRPC cells.

Importantly, compared to castration-sensitive prostate cancer (CSPC) tissues, CRPC tissues exhibited significantly higher mRNA levels of *SNRPA*, *NDUFS8*, and *NDUFS9* (Fig. 8O–Q), as well as increased ATP contents (Fig. 8R) and mtDNA levels (Fig. 8S). To investigate if *SNRPA* expression can modify the cellular response to androgen supplementation, we treated control (kdC) and SNRPA-silenced (kdSNRPA-sh1) pPC-1 cells with 1 nM of R1881, a synthetic androgen analog. We found that R1881 supplementation had no significant impact on mitochondrial function (ATP contents, Fig. S2J), proliferation (Fig. S2K) or apoptosis (Fig. S2L) in kdC pPC-1 cells. Importantly, R1881 supplementation did not significantly rescue ATP reduction (Fig. S2M), proliferation inhibition (Fig. S2N) or apoptosis induction (Fig. S2O) phenotype induced by SNRPA silencing in pPC-1 cells. These findings indicate that the impact of androgen supplementation with R1881 was minimal in pPC-1 cells and not significantly modified by SNRPA expression levels.

### Silencing of SNRPA impedes the growth of CRPC cell xenografts in a nude mice model

**Fig. 9:**
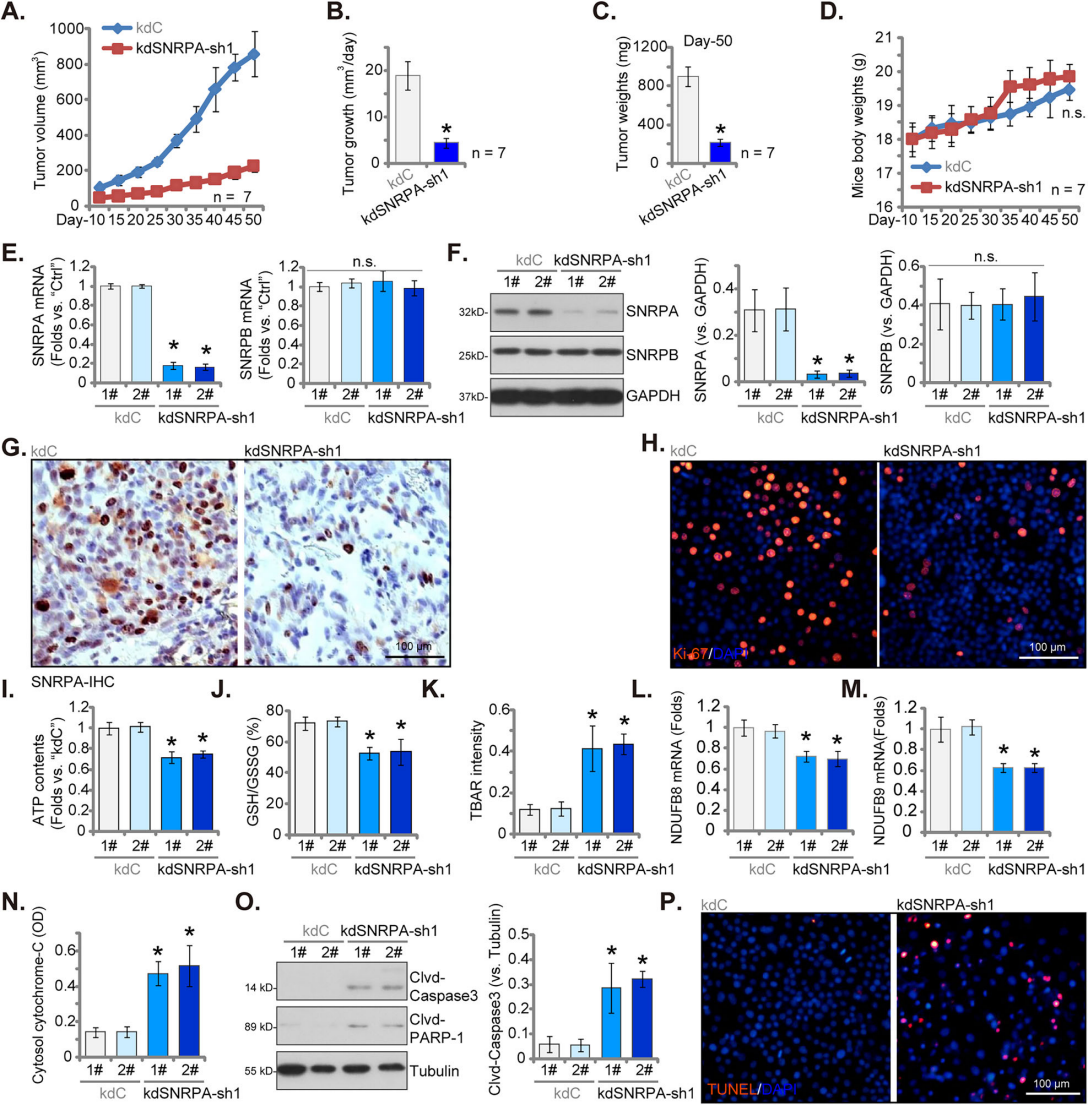
Silencing of SNRPA impedes the growth of CRPC cell xenografts in a nude mice model. Stable pPC-1 primary cancer cells expressing either SNRPA shRNA (“kdSNRPA-sh1”) or control shRNA (“kdC”) were subcutaneously implanted into the flanks of nude mice, with each animal receiving six million cells. Tumor growth monitoring commenced ten days (Day-10) post-implantation. Tumor volumes (**A**, mm³) and body weight (**D**, g) were measured every six days from Day-10 to Day-50. Daily tumor growth rates were calculated (**B**). Xenografts were excised on Day-50 and individually weighed (**C**). Two xenografts per group (1# and 2#) were subjected to the listed protein and mRNA expression profiling in tissue lysates (**E**, **F**, **L**, **M** and **O**). Cytosolic cytochrome-c levels in tissue lysates were quantified via ELISA (**N**). ATP contents (**I**), GSH/GSSG ratio (**J**) and TBAR intensity (**K**) were also measured. Immunohistochemical (IHC) staining for SNRPA was performed on tissue sections (**G**). Ki-67 (**H**) and TUNEL (**P**) fluorescence staining were conducted on tissue slices. Data are presented as mean ± standard deviation (SD). Statistical significance is indicated by *P < 0.05 compared to “kdC” group. “n.s.” denotes non-statistically significant differences (***P*** > 0.05). The study included seven mice per group for experiments **A**–**D**. For experiments **E**–**P**, each xenograft was divided into five sections for individual analysis.

To elucidate the critical role of SNRPA in CRPC cell growth in vivo, six million of pPC-1 cells transduced with either “kdSNRPA-sh1” or control shRNA (“kdC”) were subcutaneously injected into nude mice. Tumor growth was monitored commencing ten days post-injection (designated as “Day-10”). Volumetric assessments conducted every five days from Day-10 to Day-50 unveiled a substantial reduction in tumor growth within the kdSNRPA-sh1-expressing pPC-1 xenografts compared to kdC controls (Fig. 9A). Further analysis of daily growth trajectories substantiated the profound anti-proliferative effect of kdSNRPA-sh1 on CRPC tumorigenesis (Fig. 9B). At Day-50, all tumors were excised and subjected to analysis, revealing significantly diminished masses in kdSNRPA-sh1 pPC-1 xenografts relative to kdC pPC-1 xenografts (Fig. 9C). No appreciable differences in body weight were observed between the two experimental cohorts (Fig. 9D). Next, two xenografts per group (designated as 1# and 2#) were harvested at Day-50. A significant reduction in *SNRPA* mRNA and protein levels was observed in kdSNRPA-sh1-expressing pPC-1 xenograft tissues (Fig. 9E–F), while SNRPB expression remained unaffected (Fig. 9E–F). Immunohistochemistry (IHC) staining corroborated the downregulation of SNRPA protein within kdSNRPA-sh1 pPC-1 xenografts (Fig. 9G), consistent with the protein’s established nuclear localization (Fig. 9G).

Further tissue fluorescence analysis revealed a significant decrease in Ki-67 nuclear staining within SNRPA-silenced pPC-1 xenografts, indicative of suppressed in vivo proliferation (Fig. 9H). Xenograft tissues derived from pPC-1 cells expressing kdSNRPA-sh1 exhibited a significant reduction in ATP contents (Fig. 9I). Concomitantly, these tissues demonstrated an reduced GSH/GSSG ratio (Fig. 9J) and elevated TBAR intensity (Fig. 9K), indicative of impaired OXPHOS and oxidative stress. Furthermore, mRNA expression levels of NDUFB8 and NDUFS9, subunits of mitochondrial complex I, were markedly downregulated in SNRPA-silenced pPC-1 xenograft tissues (Fig. 9L–M). Conversely, a marked increase of cytosol cytochrome C (Fig. 9N) accompanied by increased levels of cleaved-Caspase-3 and cleaved-PARP-1 (Fig. 9O) were detected in kdSNRPA-sh1-expressing pPC-1 xenografts. These findings were corroborated by additional tissue fluorescence staining, which demonstrated a significantly higher proportion of TUNEL-positive nuclei in kdSNRPA-sh1 pPC-1 xenograft sections (Fig. 9P), providing compelling evidence for the induction of apoptosis. Collectively, these data unequivocally demonstrated that silencing of SNRPA impeded the growth of pPC-1 xenografts in a murine model.

## Discussion

Despite advancements in understanding the molecular underpinnings of CRPC, the development of effective targeted therapies has been hindered by the complex and heterogeneous nature of the disease [3, 18]. While initial responses to androgen deprivation therapy (ADT) and subsequent hormonal interventions are often observed, the emergence of CRPC invariably leads to therapeutic resistance [3, 18–20]. Additionally, the intricate interplay between tumor cells and the tumor microenvironment, coupled with the dynamic evolution of genomic alterations within the cancer cell population, contributes to the challenge of identifying and targeting sustained vulnerabilities [3, 18–20]. Consequently, the clinical efficacy of existing targeted therapies remains limited, necessitating the identification of novel therapeutic strategies to address the unmet clinical needs of patients with CRPC [3, 18–20].

Our findings indicate that SNRPA is a critical regulator of CRPC progression and a potential therapeutic target. Bioinformatics analyses established a positive correlation between SNRPA overexpression and prostate cancer aggressiveness, with patients exhibiting high SNRPA levels demonstrating poor clinical outcomes. Single-cell RNA sequencing data further supported the involvement of SNRPA in prostate cancer pathogenesis by revealing enriched expression within cancer cells. SNRPA is also upregulated in both locally treated CRPC tissues and a panel of CRPC cells. Functional studies demonstrated that SNRPA shRNA or KO significantly suppressed CRPC cell proliferation, migration, and induced apoptosis. Conversely, ectopic SNRPA overexpression promoted an aggressive cancer cell phenotype. In vivo, shRNA-mediated inhibition of SNRPA expression impeded the growth of subcutaneous xenografts derived from primary CRPC cells in a nude mouse model.

Tumorigenesis necessitates both genetic alterations and metabolic adaptations. While glycolysis is often upregulated in cancer, prostate cancer uniquely relies on OXPHOS. Normal prostate epithelium diverts citrate from mitochondrial OXPHOS for lipid synthesis, resulting in higher basal OXPHOS levels. This metabolic phenotype persists during tumor progression, emphasizing the importance of mitochondrial function in prostate cancer [21]. Indeed, CRPC cells often shift towards enhanced OXPHOS and ATP production to meet the increasing energy demands required for sustained growth and survival under androgen-deprived conditions [21].

**Fig. 10:**
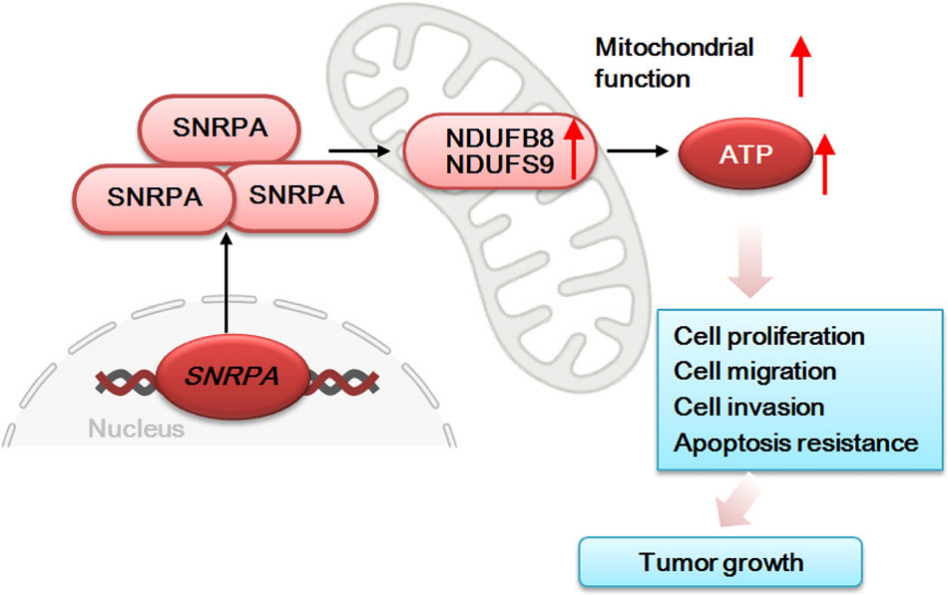
The proposed signaling carton of this study. Increased SNRPA levels in CRPC cells lead to elevated NDUFB8/NDUFS9 expression, enhanced mitochondrial function, increased ATP production, and ultimately promote various cellular processes that drive CRPC growth in vitro and in vivo.

Here, we found that SNRPA is important for OXPHOS and ATP generation in CRPC cells. Depletion of SNRPA via shRNA or KO led to a significant reduction in complex I activity, ATP synthesis, and mitochondrial membrane potential, accompanied by an increase in ROS production in primary CRPC cells. The detrimental effects of SNRPA knockdown were attenuated by ATP supplementation or the antioxidant NAC, indicating a causal relationship between SNRPA depletion, mitochondrial dysfunction, and CRPC cell death. Conversely, SNRPA overexpression enhanced mitochondrial complex I activity and ATP biosynthesis. In vivo, SNRPA knockdown in pPC-1 xenografts resulted in decreased ATP levels, impaired redox balance, as evidenced by a reduced GSH/GSSG ratio, and increased lipid peroxidation. These findings suggest that SNRPA promotes CRPC cell growth, at least in part, by augmenting OXPHOS and ATP production (see Fig. 10).

NDUFB8 and NDUFS9 are integral components of the mitochondrial complex I, playing pivotal roles in the OXPHOS and ATP synthesis. NDUFB8 is essential for the structural stability of mitochondrial complex I, facilitating efficient electron transport within OXPHOS pathway, which is crucial for ATP synthesis [22, 23]. NDUFS9 is directly involved in the electron transfer chain, facilitating the transfer of electrons from NADH to ubiquinone, a key step in driving the proton gradient necessary for ATP production [22, 23]. The present study demonstrates a critical role for SNRPA in the transcriptional regulation of NDUFB8 and NDUFB9 in CRPC cells. Both knockdown/KO and overexpression experiments revealed a direct correlation between SNRPA and the expression levels of these Complex I subunits in primary CRPC cells. Consistent findings were observed in vivo, where decreased *NDUFB8* and *NDUFB9* expression accompanied SNRPA suppression in pPC-1 xenograft models. These data collectively indicate that SNRPA-mediated enhancement of OXPHOS and ATP production in CRPC cells could be due to its upregulation of NDUFB8 and NDUFB9. These findings collectively suggest that SNRPA plays a pivotal role in CRPC progression possibly by promoting OXPHOS and ATP synthesis, and represents a promising therapeutic target.

## Supplementary information

Original Data Set

Figure S1-S2

## Data Availability

All data are in the Figures and its Supplement Files.
