# Supplementary material for: SNRPA upregulation promotes mitochondrial function and drives CRPC aggressiveness
Source: Cell Death Dis. 2025 Dec 7;17(1):74. doi: 10.1038/s41419-025-08302-8 (PMC12827962; doi:10.1038/s41419-025-08302-8)
Supplement: Supplementary file 1 — Original Data Set [file 41419_2025_8302_MOESM1_ESM.pdf]

Figure S1

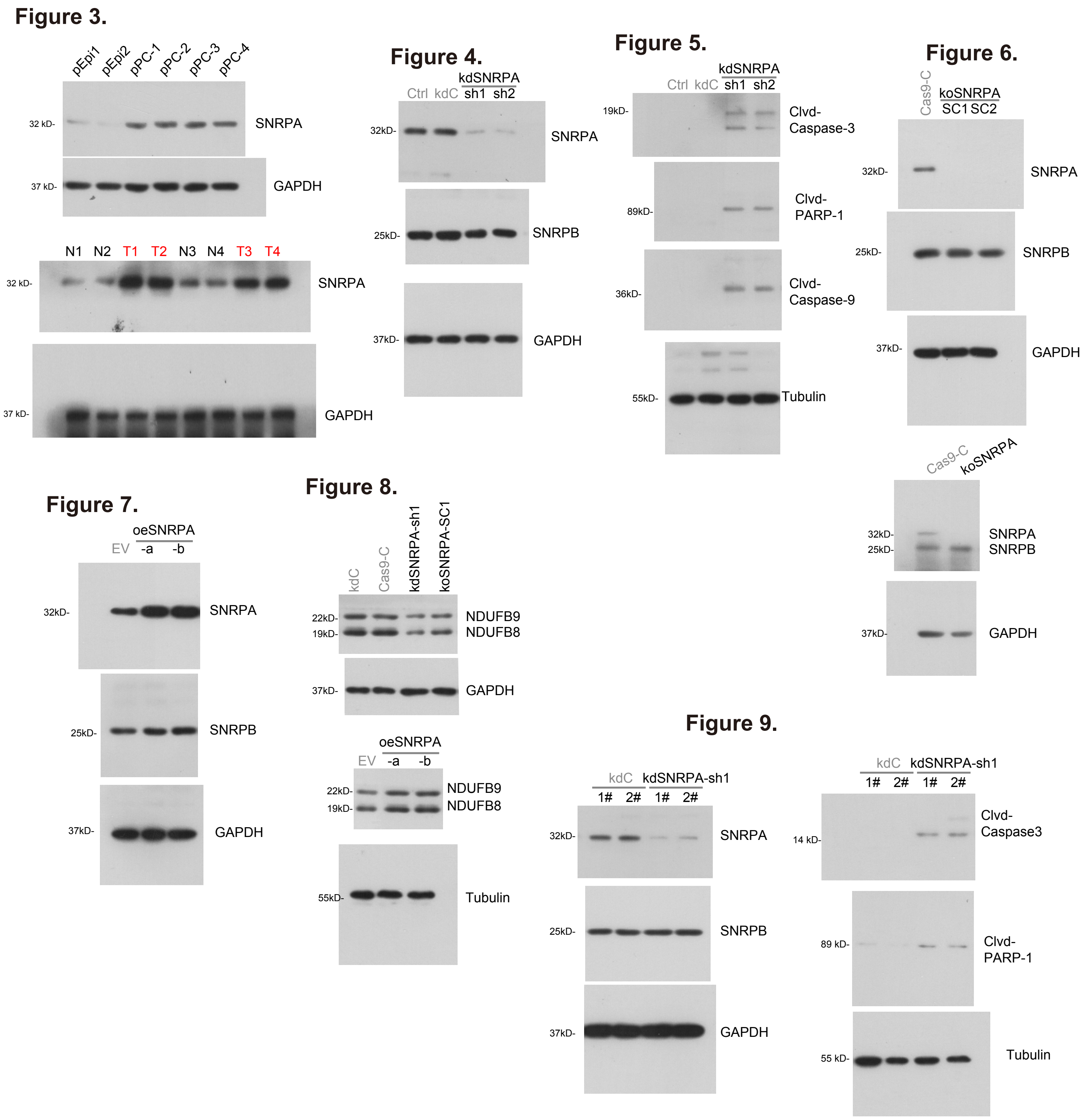

Figure S1: The uncropped blotting images of the study.
