# Supplementary material for: SNRPA upregulation promotes mitochondrial function and drives CRPC aggressiveness
Source: Cell Death Dis. 2025 Dec 7;17(1):74. doi: 10.1038/s41419-025-08302-8 (PMC12827962; doi:10.1038/s41419-025-08302-8)
Supplement: Supplementary file 2 — Figure S1-S2 [file 41419_2025_8302_MOESM2_ESM.pdf]

Figure S1

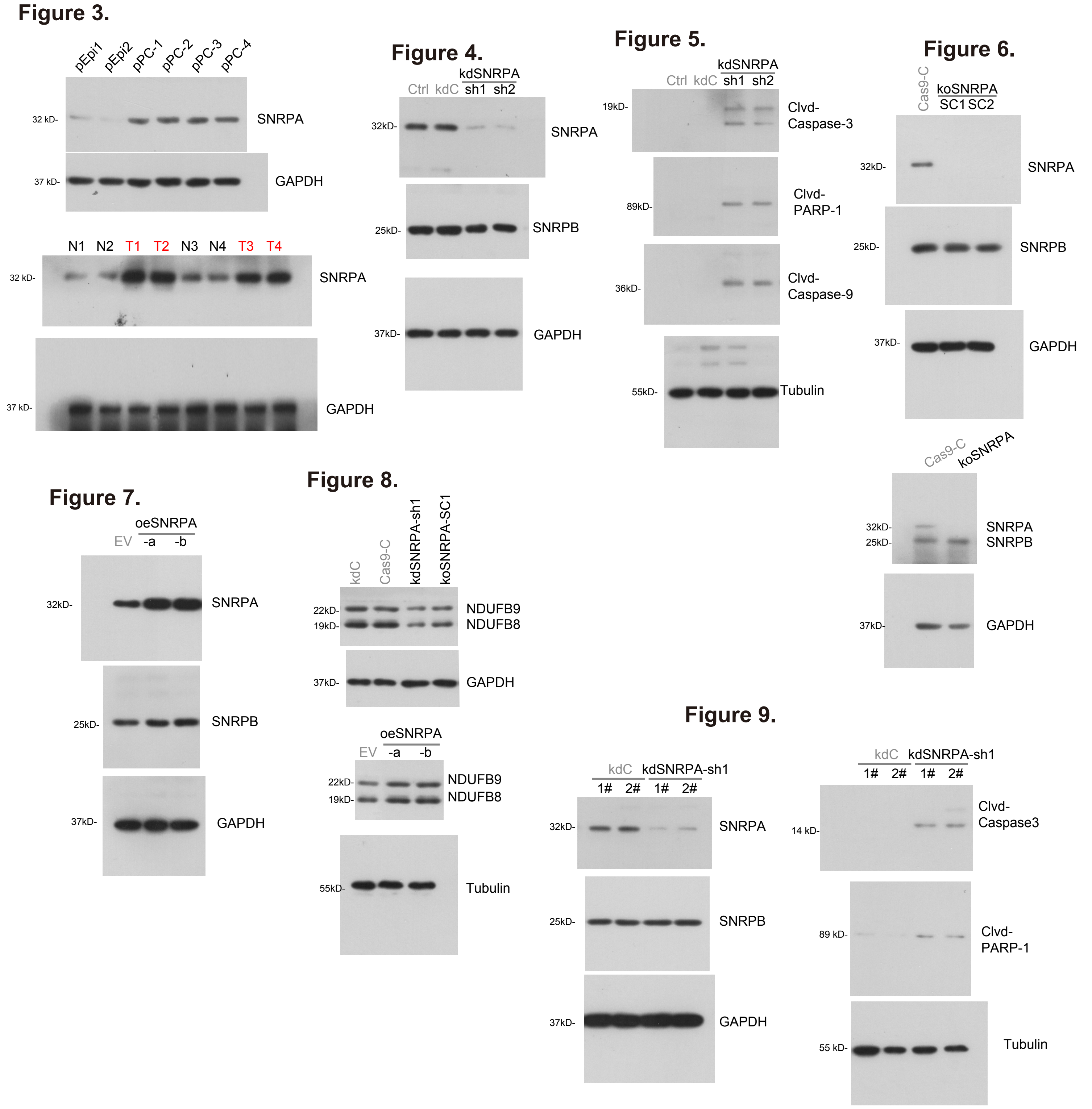

Figure S1: The uncropped blotting images of the study.

Figure S2.

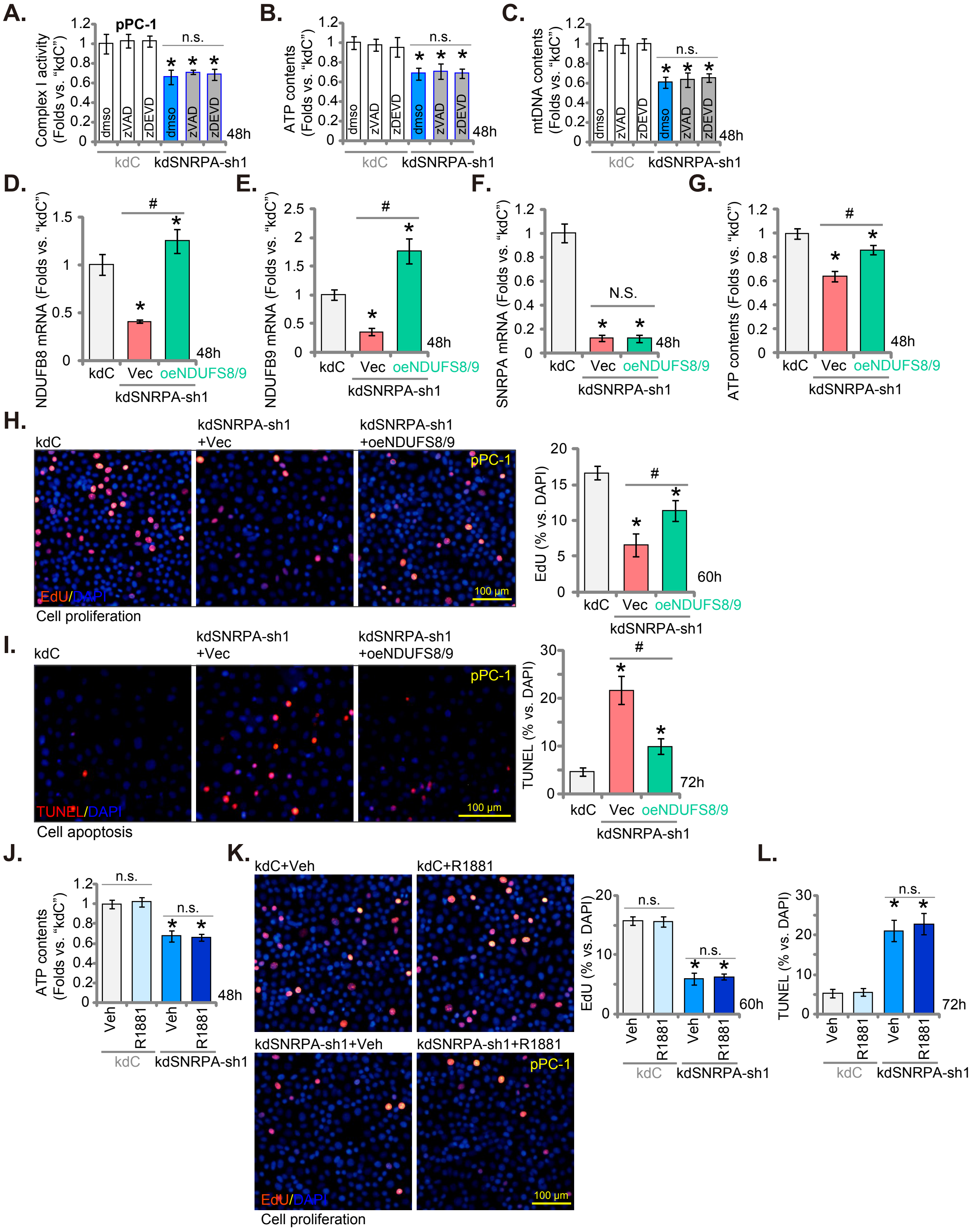
A.
B.
C.
D.
E.
F.
G.
H.
I.
J.
K.
L.
pPC-1
Complex I activity (Folds vs. "kdC")
ATP contents (Folds vs. "kdC")
mtDNA contents (Folds vs. "kdC")
dmso
zVAD
zDEVD
n.s.
48h
kdC
kdSNRPA-sh1
NDUFB8 mRNA (Folds vs. "kdC")
NDUFB9 mRNA (Folds vs. "kdC")
SNRPA mRNA (Folds vs. "kdC")
ATP contents (Folds vs. "kdC")
N.S.
#
Vec
oeNDUFS8/9
kdSNRPA-sh1 +Vec
kdSNRPA-sh1 +oeNDUFS8/9
EdU/DAPI
100 μm
Cell proliferation
EdU (% vs. DAPI)
60h
TUNEL/DAPI
Cell apoptosis
TUNEL (% vs. DAPI)
72h
Veh
R1881
kdC+Veh
kdC+R1881
kdSNRPA-sh1+Veh
kdSNRPA-sh1+R1881

Figure S2. pPC-1 cells expressing either kdC or kdSNRPA-sh1 were treated with zVAD-fmk (zVAD, 50 μM), zDEVDfmk (zDEVD, 50 μM) or vehicle control (0.1% DMSO, “dmso”) for 48h, the mitochondrial complex I activity (**A**), ATP (**B**) and mtDNA contents (**C**) were tested. The kdSNRPA-sh1-expressing pPC-1 cells were further transduced with the NDUFS8-expressing and NDUFS9-expressing lentiviral constructs ("oeNDUFS8/9"), stable cells were formed and expression levels of listed mRNAs were shown (**D**-**F**); Cells were further were cultured for defined time points, ATP contents (**G**), cell proliferation (by measuring nuclear EdU incorporation, **H**) and apoptosis (via TUNEL staining assays, **I**) were measured. pPC-1 cells expressing either kdC or kdSNRPA-sh1 were treated with a potent synthetic androgen R1881 (1 nM) for designated hours, ATP contents (**J**), cell proliferation (by measuring nuclear EdU incorporation, **K**) and cell apoptosis (via TUNEL staining assays, **L**) were tested. Data are presented as mean ± standard deviation (SD) with n=5 biological replicates. Statistical significance is indicated by $^{*}P < 0.05$ compared to "kdC" cells. $^{\#}P < 0.05$ (**D**-**I**). "n.s." denotes non-statistically significant differences ($P > 0.05$). Consistent results were obtained across all five biological replicates. The scale bar in microscope images represents 100 μm.
